# Immune Checkpoint Inhibitor–Mediated Aseptic Meningitis and Hypophysitis

**DOI:** 10.1155/crom/3517328

**Published:** 2025-03-08

**Authors:** Pavel Bleik, Panisara Fangsaard, Nataliya Yuklyaeva

**Affiliations:** Department of Internal Medicine, Bassett Medical Center, Cooperstown, New York, USA

**Keywords:** immunotherapy, ipilimumab, nivolumab, oncology, renal cell carcinoma

## Abstract

Immune checkpoint inhibitors have revolutionized cancer treatment, yet their use is associated with unique and sometimes unpredictable immune-related adverse events. We present a case of a 67-year-old female with renal cell cancer treated with ipilimumab and nivolumab who developed aseptic meningitis and hypophysitis. This case highlights the challenges in managing immune-related adverse events and underscores the need for vigilance in monitoring patients receiving ICIs.

## 1. Case Report

A 67-year-old woman presented to the oncology clinic for evaluation of anemia. Her medical history included sigmoid diverticulosis, Type 2 diabetes mellitus, hypertension, and internal hemorrhoids. She was independent in activities of daily living, a former smoker who quit many years ago, and denied alcohol consumption.

The patient complained of weakness and heart palpitations, prompting evaluation in a cardiology clinic. An EKG revealed sinus tachycardia with a heart rate of 106 bpm. Transthoracic ECHO showed no abnormalities, but due to persistent tachycardia, metoprolol was initiated. Over the past 3 years, she had experienced an unintentional weight loss of 20 pounds, which she attributed to metformin prescribed for diabetes management. At the time of the appointment, her hemoglobin was 9.4 g/dL (*n* = 11.5–15.5 g/dL), hematocrit was 31.4% (*n* = 34.0%–46.0%), MCH was 23.9 pg (*n* = 27.0–33.5 pg), MCHC was 29.9 g/dL (*n* = 31.5–35.5 pg), and MCV was 79.9 fL (*n* = 80–100 fL), with other peripheral blood cell lines within normal limits. Inflammatory markers were elevated with an ESR of 87 mm/hr and CRP of 10.7 mg/L. Reticulocyte hemoglobin equivalent was inappropriately low for the degree of anemia (24.3 pg) with a reticulocyte production index of 0.5. Physical examination identified a firm left flank mass; SPEP did not show an M-spike, and the hemolysis workup was negative.

**Figure 1:**
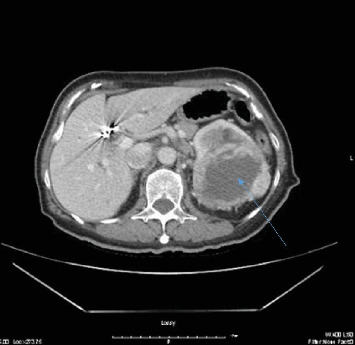
Left renal mass (*the blue arrow*) measured 10 × 8 × 10 cm clinically correlates with malignancy.

The patient underwent a CT scan, which revealed a large renal mass measuring 10 × 8 × 10 cm. Additionally, multiple pulmonary nodules and subcarinal and retroperitoneal adenopathy were noted, all consistent with metastatic malignancy (Figure 1).

**Figure 2:**
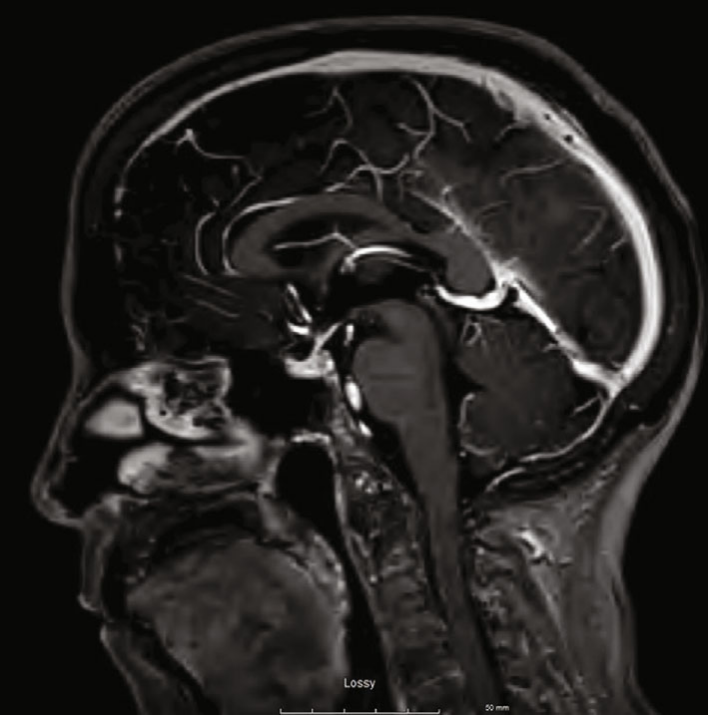
Brain MRI done before the treatment was started. No evidence of metastatic disease. Hypophysis appears normal.

A month following diagnosis, the patient underwent a left radical nephrectomy, and postoperative recovery was uneventful. Histopathological examination confirmed Stage IV metastatic renal cell carcinoma originating from the left kidney. Imaging studies, including MRI, ruled out brain metastasis but incidentally noted mild paranasal sinus disease (Figure 2).

Prior to starting ipilimumab and nivolumab for metastatic renal cell carcinoma, the patient underwent comprehensive blood testing. Results revealed normal electrolyte levels with sodium at 137 mEq/L (*n* = 136–145 mEq/L), potassium at 4.4 mEq/L (*n* = 3.5–5.1 mEq/L), and chloride at 104 mEq/L (*n* = 98–110 mEq/L). Glucose was at 110 mg/dL (*n* = 70–139 mg/dL). Renal function tests indicated a BUN level of 29 mg/dL (*n* = 7–25 mg/dL) and creatinine within normal limits at 1.1 mg/dL (*n* = 0.6–1.2 mg/dL).

The liver profile showed alkaline phosphatase levels at 112 U/L (*n* = 7–72 U/L), with normal transaminase levels. Endocrine evaluations included ACTH at 25 pg/mL (*n* = 7.2–63 pg/mL) and TSH at 2.37 mIU/L (*n* = 0.41–4.5 mIU/L), both within normal ranges. Hematologic parameters showed hemoglobin at 11 g/dL, RBC at 4.24 × 10^6^/*μ*L (*n* = 3.7–5.1, 24 × 10^6^/*μ*L), MCH at 25.9 pg, RDW at 50.1% (*n* = 37.3–49 fL), and MCHC at 30.9 g/dL.

Three weeks into treatment, the patient presented for a follow-up appointment reporting tolerable side effects, including transient scalp, neck, and armpit itchiness and dryness that had resolved. Her blood glucose was slightly elevated at 180 mg/dL, blood pressure was 128/80 mmHg, and pulse was 87 bpm. Treatment was continued as planned.

Two weeks later, during a routine follow-up visit, her renal function showed a slight decline with a creatinine level of 1.6 mg/dL and elevated potassium at 5.0 mEq/L, alongside mild hyponatremia of 131 mEq/L. Other laboratory parameters, including CBC, BMP, ACTH, and protein levels, remained within normal limits. Dapagliflozin was introduced due to rising creatinine levels. Metformin was stopped.

One month later, during another follow-up appointment, the patient reported an episode of transient double vision that resolved spontaneously. Her creatinine levels had improved to 1.3 mg/dL. Thyroid function tests indicated a decrease in TSH to 0.39 mIU/L (*n* = 0.41–4.5 mIU/L), with thyroid hormone levels showing T4 at 0.5 ng/dL (*n* = 0.6–1.3 5 ng/dL) and T3 at 40 ng/dL (*n* = 79–149 ng/dL). A slight elevation in WBC count was noted but was clinically insignificant.

Two days following this visit, the patient was admitted to the hospital with altered mental status. She was found by her family in the morning, confused with a left shoulder droop. She experienced hypotension and tachycardia, alongside blurry vision and swelling of the left eye. Physical examination revealed a limited neck range of motion, although she denied headache, shortness of breath, cough, nausea, vomiting, or diarrhea.

Physical examination was notable for altered mental status, left ptosis, left cranial nerve VI weakness, nuchal rigidity, and submandibular fullness. Vital signs showed a BP of 113/66 mmHg, pulse of 117 bpm, and temperature of 37.2°C.

Initial laboratory tests revealed a mild elevation in troponin at 29.5 ng/mL (*n* = <15 ng/mL), along with electrolyte abnormalities including sodium 131 mEq/L, potassium 4.6 mEq/L, and chloride 96 mEq/L. Renal function showed a creatinine level of 1.9 mg/dL and elevated alkaline phosphatase at 118 U/L (*n* = 34–104 U/L), AST at 64 U/L (*n* = 13–39 U/L), and cortisol at 5.2 mcg/dL (*n* = 6–27 mcg/dL). WBC count was elevated at 11.4 × 10^9^/L (*n* = 3.7–10.6, 4 × 10^9^/L), with ACTH elevated at 60 pg/mL, TSH at 0.63 mIU/L, and T3 at 2.3 ng/mL. Other hormonal assays, including FSH, LH, IGF-1, and prolactin, were normal. All other laboratory values were within normal ranges. Herpes and viral panel were negative.

**Figure 3:**
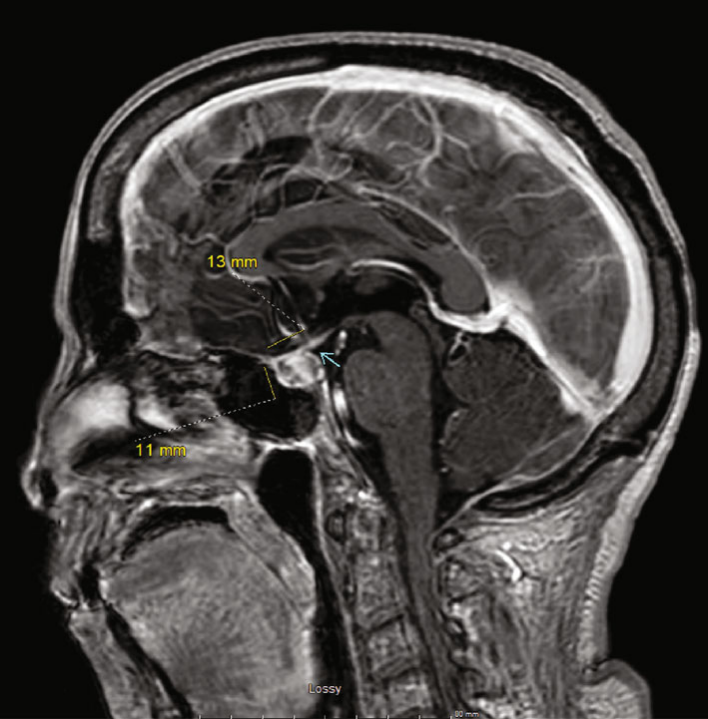
MRI done during the hospital admission. The blue arrow points to changes in the pituitary gland.

Suspected diagnoses included meningitis, cavernous sinus thrombosis, and hypophysitis. Treatment initiated included meropenem, vancomycin, hydrocortisone, IV fluids, and heparin drip. A blood culture was obtained, and a lumbar puncture was performed, which yielded colorless CSF; opening pressure was normal. CSF analysis revealed predominantly lymphocytic pleocytosis (WBC of 35 cells/mm^3^ and lymphocytes of 87%) and elevated protein (72 mg/dL). A subsequent brain MRI demonstrated a new heterogeneous enhancement of the pituitary gland, indicative of hypophysitis, with loss of normal hyperintense T1 signal. Cavernous sinus enlargement was not observed (Figure 3).

**Figure 4:**
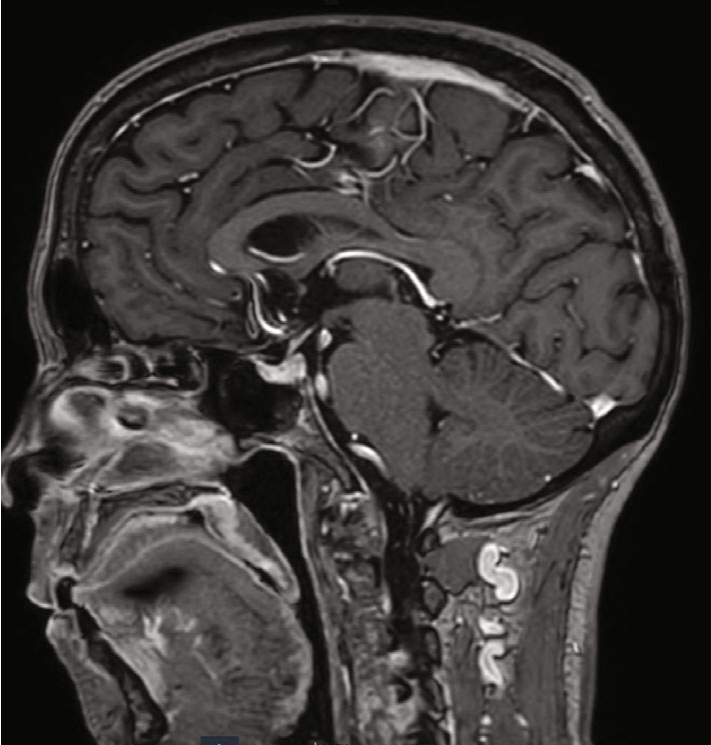
MRI done 4 weeks later shows no abnormalities.

Following negative blood cultures and testing negative for infectious agents including *Cryptococcus* spp., influenza, HSV, and COVID-19, the patient showed improvement 3 days later and was discharged with plans for a follow-up MRI in 4 weeks. Upon discharge, her laboratory values included stable electrolytes with sodium at 136 mEq/L, potassium at 4.4 mEq/L, and improved renal function with creatinine at 1.0 mg/dL. IgG was at 973 mg/dL (*n* = 635–1.741 mg/dL), and ACTH and TSH levels were within normal limits. All other blood work was unremarkable. She was discharged on a regimen including hydrocortisone for ongoing management of hypophysitis and continued medications including metformin, dapagliflozin, glimepiride, lisinopril, metoprolol, and rosuvastatin. A subsequent MRI performed 4 weeks later demonstrated complete resolution of the inflammatory changes in the pituitary gland, with an essentially unremarkable appearance. The previously noted enlargement and increased enhancement of the pituitary gland had resolved, indicating a successful treatment response and resolution of hypophysitis (Figure 4).

Following the resolution of hypophysitis confirmed by normal MRI findings and stable laboratory parameters, including ACTH, TSH, T4, LH, FSH, and WBC count, the patient's treatment regimen was adjusted. Ipilimumab was discontinued, while nivolumab was temporarily held for 2 weeks and then cautiously resumed. At the latest follow-up, there have been no reported adverse effects from the resumption of nivolumab.

## 2. Discussion

While immunotherapy-related hypophysitis and endocrinopathies are quite common, meningitis and encephalitis are relatively rare but notable side effects of immune checkpoint inhibitors [1]. A case series study spanning from 2015 to 2019 highlighted several instances where these neurological complications presented variably across different patients. For instance, a 46-year-old female receiving ipilimumab for Stage IV melanoma exhibited symptoms including headaches, hearing loss, dizziness, and low-grade fever. Her CSF analysis showed a high lymphocyte count with negative flow cytometry, alongside MRI findings consistent with hypophysitis. Treatment involved intravenous methylprednisolone followed by oral dexamethasone with subsequent tapering. Similarly, a 70-year-old male undergoing nivolumab therapy for Stage IV renal cell carcinoma presented with neck pain, stiffness, fever, confusion, gait disturbances, and dysphasia. MRI confirmed signs of ventriculitis, and CSF analysis mirrored lymphocytic pleocytosis seen in other cases. He received steroid therapy, including dexamethasone, followed by methylprednisolone and oral prednisolone over 6 months [2]. These cases underscore the diverse clinical manifestations and treatment challenges associated with immunotherapy-related central nervous system complications.

In contrast to the cases described in prior studies, our patient presented with a distinct constellation of symptoms, including visual disturbances, high-grade fever persisting for several days, confusion, and clinical signs indicative of hypophysitis with associated thyroid and adrenal insufficiency, despite normal ACTH levels. As it was previously reported, this patient had demonstrated classical symptoms of pituitary insufficiency including suppressed TSH level. Although ACTH level was initially normal, a month after the admission it had decreased and remained suppressed a year after, possibly indicating an acute inflammatory phase with ACTH-producing cell initial destruction and ACTH release. Also, it might have been explained by a decreased cortisol level in the settings of possible adrenal insufficiency [3]. Similar to the cases mentioned, our patient's lumbar puncture findings and MRI features were consistent with aseptic meningitis. Treatment with broad-spectrum antibiotics was started empirically until CSF analysis became available. Acyclovir was not initiated because HSV testing had been done on the first day of admission and came back negative by the time the treatment was started. This underscores the variability in neurological complications associated with immunotherapy, which can manifest with diverse clinical presentations that may mimic infectious processes or other conditions.

Nivolumab is a monoclonal IgG4 antibody that targets the anti-PD-1 (programmed death-1) receptor with high specificity and affinity. PD-1 receptor is expressed on T-cells and dampens their immune response by binding ligands PD-L1 and PD-L2 on antigen-presenting cells (APCs) [4]. In a cancer state, PR-L1 expression by tumor cells or immune cells in the tumor environment deactivates PD-1-expressing tumor-infiltrating lymphocytes and facilitates tumor cells to escape the immune recognition and elimination phenomenon [5, 6].

Ipilimumab is a cytotoxic T lymphocyte antigen-4 (CTLA-4) monoclonal antibody [7]. There are two signals necessary for T-cell activation. The first one is major histocompatibility complex (MHC) I and II receptors on T-cells binding to tumor-associated antigens (TAAs) presented by APCs. The second one is the CD-28 receptor on T-cells binding to CD-80 and CD-86 on APCs. These two mechanisms result in T-cell proliferation and cytokine release, triggering the immune response. Through this process, being occurred CTLA-4 becomes upregulated, competing with CD-28 for CD-80 and CD-86 binding on APCs. Ipilimumab prevents CD-80 and CD-86 on APCs from binding CTLA-4 on T-cells. This blockage allows for T-cell activation and proliferation, amplifying T-cell-mediated immunity and augmenting the host's immune system response to the tumor [8].

ICP toxicity is driven by excessive T-cell activation, mimicking autoimmune states, which can affect any organ system [9]. Diverse immune cells, especially T lymphocytes, populate the meninges under healthy conditions, as well as in inflammatory and neurodegenerative states [10]. For example, T-cell-mediated mechanisms might be responsible for antibody production to the nuclear or cytoplasmic antigens in immune-mediated encephalitis. T-cell responses to HuD have been demonstrated in patients with paraneoplastic encephalomyelitis [11]. Brain or peripheral nerve tissues from these patients show more infiltration by T lymphocytes than by B lymphocytes [12, 13], and T-cells are found in close contact with neurons that express MHC Class I molecules [14].

While morphological studies were not conducted, it is hypothesized that T-cell infiltration into the meninges, hypophysitis, and involvement of the thyroid and adrenal glands may underlie the symptoms observed in our patient.

## 3. Conclusion

Immunotherapy-mediated hypophysitis is a well-documented and frequently reported complication, contrasting with the less common occurrences of encephalitis or meningitis. When patients present with symptoms resembling these complications, it is crucial to consider the possibility of immune-related adverse events, as they can mimic more prevalent conditions such as infectious processes. Early recognition and prompt management of potential immunotherapy-related complications are essential. This includes appropriate adjustment of the treatment regimen and proactive patient education, despite their rarity, to ensure timely intervention and optimize patient outcomes.

## Data Availability

The data supporting the findings of this case report are available from the corresponding author upon reasonable request. All relevant clinical and laboratory data are included within the manuscript and its supporting information. Due to patient confidentiality and privacy concerns, patient-specific data cannot be shared publicly.

## Nomenclature

ACTH: adrenocorticotropic hormone
TSH: thyroid-stimulating hormone
T4: thyroxine
T3: triiodothyronine
RBC: red blood cell count
PD: program death
LH: luteinizing hormone
FSH: follicle-stimulating hormone
MRI: magnetic resonance imaging
HSV: herpes simplex virus
ALT: alanine transaminase
AST: aspartate transaminase
BUN: blood urea nitrogen
MCHC: mean corpuscular hemoglobin concentration
MCH: mean corpuscular hemoglobin
RDW: red cell distribution width
CN: cranial nerve
EKG: electrocardiogram
ECHO: echocardiogram
TIBS: total iron binding capacity
CBC: complete blood count
BMP: basic metabolic panel
SOB: shortness of breath
Hb: hemoglobin
MHC: major histocompatibility complex
BP: blood pressure
ESR: erythrocyte sedimentation rate
CRP: C-reactive protein
SPEP: serum protein electrophoresis
CT: computed tomography
WBC: white blood cell
IGF-1: insulin-like growth factor 1
IgG: immunoglobulin G
APC: antigen-presenting cell
CTLA: cytotoxic T lymphocyte–associated antigen
CSF: cerebrospinal fluid
